# Higher sclerostin is associated with pulmonary hypertension in pre-dialysis end-stage kidney disease patients: a cross-sectional prospective observational cohort study

**DOI:** 10.1186/s12890-024-02871-8

**Published:** 2024-02-10

**Authors:** Jonghyun Lee, Dong-Hyuk Cho, Hyeon-Jin Min, Young-Bin Son, Tae Bum Kim, Se Won Oh, Myung-Gyu Kim, Won Yong Cho, Sang-Kyung Jo, Jihyun Yang

**Affiliations:** 1 Department of Internal Medicine, Korea University Anam Hospital, Koreadae-Ro 73, Sungbuk-Gu, Seoul, Korea; 2 Division of Nephrology, Department of Internal Medicine, Sungkyunkwan University School of Medicine, Kangbuk Samsung Hospital, 29 Saemunan-ro, Jongno-gu, 03181 Seoul, Korea

**Keywords:** Chronic kidney disease, End-stage kidney disease, Mortality, Pulmonary hypertension, Wnt signaling

## Abstract

**Background:**

Pulmonary hypertension (PH) is a complication of chronic kidney disease (CKD) that contributes to mortality. Sclerostin, a SOST gene product that reduces osteoblastic bone formation by inhibiting Wnt/β-catenin signaling, is involved in arterial stiffness and CKD-bone mineral disease, but scanty evidence to PH. This study explored the relationship between sclerostin and PH in CKD 5, pre-dialysis end-stage kidney disease (ESKD) patients.

**Methods:**

This cross-sectional prospective observational cohort study included 44 pre-dialysis ESKD patients between May 2011 and May 2015. Circulating sclerostin levels were measured using an enzyme-linked immunosorbent assay. PH was defined as an estimated pulmonary artery systolic pressure > 35 mmHg on echocardiography.

**Results:**

Patients with higher sclerostin levels ≥ 218.18pmol/L had echocardiographic structural cardiac abnormalities, especially PH (*P* < 0.01). On multivariate logistic analysis, sclerostin over 218.19pmol/L was significantly associated with PH (odds ratio [OR], 41.14; 95% confidence interval [CI], 4.53-373.89, *P* < 0.01), but multivariate Cox regression analysis showed the systemic vascular calcification score over 1 point (Hazard ratio [HR] 11.49 95% CI 2.48–53.14, *P* = 0.002) and PH ([HR] 5.47, 95% CI 1.30-23.06, *P* = 0.02) were risk factors for all-cause mortality in pre-dialysis ESKD patients.

**Conclusions:**

Serum sclerostin and PH have a positive correlation in predialysis ESKD patients. The higher systemic vascular calcification score and PH have an association to increase all-cause mortality in pre-dialysis ESKD patients.

**Supplementary Information:**

The online version contains supplementary material available at 10.1186/s12890-024-02871-8.

## Introduction

Vascular calcification contributes to increased mortality in chronic kidney disease (CKD) and end-stage kidney disease (ESKD). Oxidative stress, chronic inflammation, endothelial dysfunction, and CKD–mineral and bone disorder (CKD-MBD) in CKD accelerate vascular calcification and structural arterial stiffening, increasing the cerebro/cardiovascular (CV) risk [1]. The relationship between CKD and chronic cardiac dysfunction is known as cardiorenal syndrome type 4 [2]. Along with cardiorenal syndrome, pulmonary hypertension (PH), characterized by increased blood pressure in the pulmonary arteries, has been arising as a main comorbidity in CKD. Several recent studies demonstrated that the prevalence of PH is significantly higher in patients with CKD and is associated with increased mortality and CV events [3, 4].

Sclerostin-secreted glycoprotein is a negative regulator of the Wnt signaling pathway, a potent regulator of bone metabolism; thus, it could be a novel candidate for the bone vascular axis in CKD. Although sclerostin inhibits vascular calcification, its role in vascular calcification and the clinical prognosis of CKD remains elusive. Krishna et al. reported that sclerostin inhibits aortic aneurysms and atherosclerosis development and it could be a treatment option for vascular disease [5]. At the same time, Yang et al. reported the inverse association of sclerostin and aortic calcification in long-term hemodialysis patients and they suggested higher sclerostin with less cardiovascular events (hazard ratio 0.982 per 1pmol/L increases of each sclerostin [6]. Meanwhile, Morena et al. reported sclerostin is independently associated with coronary artery calcification in non-dialysis CKD patients [7]. Sclerostin is reportedly expressed in the bone as well as in other tissues and organs, such as smooth muscle cells of the vasculature and lungs. Attenuation and deregulation of the B-natriuretic protein (BNP) and Wnt pathways are very well described in PH, a condition in which increased pulmonary vascular resistance leads to vascular wall remodeling with narrowing or occlusion of the vessel lumen and ultimately right heart failure [8]. A recent study demonstrated that sclerostin was the most upregulated gene in a microarray analysis of human pulmonary artery microdissection samples in patients with PH and that sclerostin stimulates pulmonary arterial endothelial cells in a WNT-dependent manner, suggesting a potential role of sclerostin in the pathogenesis of PH [9].

Vascular calcification frequently occurs in patients with CKD, and its incidence increases across CKD stages. Enhanced sclerostin expression was detected in calcified smooth muscle cells as well as in a mouse model of medial calcification. Evidence suggests that increased sclerostin levels may be positively associated with aortic calcification, abnormal intima-media thickness, and carotid plaques in type 2 diabetes [10]. In CKD, sclerostin levels are elevated and associated with CKD-MBD, especially in patients with high bone turnover disease and vascular calcification [10, 11]. Moreover, the increased sclerostin expression in calcified vessels in CKD animal studies was also reported [12, 13]. If sclerostin is related to changes in vasculature, it could be possible to expand whether the correlation with pulmonary arterial hypertension can be predicted. This study aims to understand the role of sclerostin in advanced impending CKD patients, especially patients never exposed to hemodialysis, which is a great hemodynamic stressful risk factor, advancing vascular calcification. Here, we assessed whether alterations in the physiology of the mineral bone disease axis due to CKD affect PH. We also examined the association between sclerostin, vascular calcification, and PH in pre-dialysis end-stage kidney disease (ESKD) patients.

## Materials and methods

### Patients and study design

This is a cross sectional prospective observational cohort study, the last follow-up date 31th December 2019. Patients visited the outpatient clinic monthly; 4 patients were missing during follow-up period. Between May 2011 and May 2015, 44 pre-dialysis ESKD patients were enrolled who plan to initiate peritoneal dialysis (PD). The blood sampling was preceding the PD catheter insertion operation. They provided written informed consent were included in this study. The exclusion criteria were parathyroidectomy, uncontrolled systemic illness such as malignancy, AIDS, advanced liver disease, and no history of echocardiography. Patient baseline characteristics, including age, sex, and history of hypertension, diabetes, coronary artery disease, and stroke, were recorded. We collected patients’ echocardiographic findings, the new-CV events and mortality. A new-CV event was defined as hospitalization with at least one of the following; percutaneous angiography with stent insertion, coronary artery bypass graft surgery, heart failure management using inotropes. All-cause mortality events were collected including sepsis, myocardial infarction, cardiac arrest, hematochezia. This prospective observational study was approved by the Korea University Ethics approval and consent to participate. (IRB no. AN11093-001).

### Biochemical analyses

The serum before PD catheter insertion operation were collected. The levels of creatinine, albumin, total cholesterol, low-density lipoprotein cholesterol, alkaline phosphatase, calcium, phosphate, intact parathyroid hormone, 25(OH)Vit D, high-sensitivity C-reactive protein, and hemoglobin were measured using the Beckman AU® 5821 chemistry analyzer (Beckman Coulter, Brea, CA, USA) according to the manufacturer’s instructions. Serum sclerostin levels were measured using an ELISA kit (Quantikine® ELISA, Human SOST Immunoassay; catalog number DSST00; R&D Systems Inc., Minneapolis, MN, USA, detectable range 15.6–1,000 pg/mL).

### Simple vascular calcification score

Plain radiographs of both hands and the pelvis were obtained, and the vascular calcification score was calculated using the Adragao scoring system [14]. The presence or absence of linear calcification in each section was scored as 1 or 0, respectively. The vascular calcification score was the sum of all sections for a total of 0–8.

### Echocardiography

According to the European Society of Cardiology/European Respiratory Society guidelines, PH is defined as an increase in the mean pulmonary artery ≥ 25 mmHg at rest as assessed by right heart catheterization [15]. However, PH can also be defined as a systolic pulmonary arterial pressure > 35 mmHg on cardiac Doppler ultrasound [16]. PH was defined as an increased pulmonary artery systolic pressure of > 35 mmHg calculated using the peak velocity of tricuspid regurgitation from the low parasternal long-axis view. We used the calculation formular; mean pulmonary artery pressure = 4(pulmonary regurgitation peak velocity)^2^ + right arterial pressure. Patients took echocardiography when they first hospitalized for PD catheter insertion operation. Conventional and two-dimensional (2D) speckle-tracking echocardiography scans were obtained for all patients. Chamber quantification was performed using 2D echocardiography images [17]. The left ventricular (LV) mass index (LVMI) and left atrial volume index were calculated using the formula recommended by the American Society of Echocardiography. The LV ejection fraction was calculated using the Simpson biplane method. The mitral peak velocities of the early and late filling (E and A) were measured on the apical four-chamber view. The early and late diastolic mitral annular velocities of the septal mitral annulus (e and a) were obtained using tissue Doppler imaging [18]. PH was defined as an increased pulmonary artery systolic pressure of > 35 mmHg calculated using the peak velocity of tricuspid regurgitation from the low-parasternal long-axis view. The LV global longitudinal strain was calculated as the mean longitudinal peak negative strain from 18 segments. A cardiologist reviewed the echocardiography without the patient’s information.

### Statistical analysis

All analyses and calculations were performed using SPSS Statistics for Windows version 26 (IBM, Armonk, NY, US). Data are presented as mean ± standard deviation (SD) or median (interquartile range) for continuous covariates or number and percentage for categorical covariates according to the distribution. Continuous variables were analyzed using Student’s t-test for normally distributed data or the Mann–Whitney test for non-normally distributed data. The chi-square test was used to analyze categorical variables. Variables with three or more groups were analyzed using Bayesian analysis of variance (JZS). In the multivariate analysis, a binary logistic regression backward model and multivariate Cox regression were used. Spearman’s correlation coefficient was used to determine the correlation between serum sclerostin and PH. Kaplan–Meier method was used for survival analysis. Statistical significance was set at *p* < 0.05.

## Results

### Patient characteristics according to sclerostin level

The patients’ baseline characteristics and laboratory data are shown in Table 1. The median follow-up period was 54.8 months (range, 1-100 months). The median age was 54 ± 15 years (range, 27–91 years), and the median sclerostin patients were divided into two groups according to sclerostin level using a receiver operating characteristic curve to determine the best-associated criterion value of sclerostin with pulmonary hypertension (Supplementary Fig. 1). Based on a cut-off value of 218.18 pmol/L, 31 patients were in the low sclerostin group, and 13 patients were in the high sclerostin group. There was characteristically no significant difference between two groups. (Table 1). All patients with hypertension, diabetes, and coronary artery disease patients were considered therapeutically well controlled including tolerable BP, HbA1c. There was no statistically significant correlation between SVCS and sclerostin level (data not shown).

**Table 1:** Patient characteristics according to the sclerostin

Variables	Low Sclerostin (< 218.18pmol/L) *n* = 31	High Sclerostin (≥ 218.18pmol/L) *n* = 13	P
Male	18(58.1%)	11(84.6%)	0.08
Age	52.2 ± 16.2	57.6 ± 13.7	0.22
Diabetes mellitus	11(35.5%)	8(61.5%)	0.10
Hypertension	28 (90.3%)	13(100%)	0.34
Coronary artery disease	25 (80.6%)	10 (76.9%)	0.54
BMI (kg/m^2^)	24.7 ± 4.3	26.7 ± 3.6	0.08
BSA (m^2^)	1.7 ± 0.2	1.7 ± 0.2	0.15
Baseline PWV (per 10 cm/min)	1719.3 ± 456	1688.5 ± 362.4	0.42
SBP (mmHg)	140 [110–170]	135 [110–152]	0.26
DBP (mmHg)	80[66–115]	80[70–100]	0.27
iPTH (pg/mL) [15–80]	204.6 ± 128.0	261.1 ± 186.3	0.13
25-OH-Vit.D (ng/mL) [20–40]	7.0 ± 5.6	5.1 ± 2.9	0.13
Hb (g/dL) [11–16]	9.2 ± 1.9	9.2 ± 0.8	0.47
Total CO2 (mg/dL) [23–29]	19.7 ± 6.6	20.4 ± 5.2	0.38
Albumin (mg/dL) [3.4–5.4]	3.4 ± 0.4	3.3 ± 0.6	0.32
ALP (U/L)[44–147]	89.7 ± 34.0	78.4 ± 21.4	0.14
Ca (mg/dL) [8.6–10.3]	7.5 ± 1.2	8.0 ± 0.8	0.09
P (mg/dL) [2.5–4.5]	5.3 ± 2.5	5.0 ± 1.6	0.33
Total Cholesterol (mg/dL) [< 200]	149.1 ± 45.7	133.2 ± 37.3	0.14
LDL (mg/dL) [< 160]	90.8 ± 34.8	81.8 ± 25.2	0.20
CRP (mg/dL) [< 0.3]	81.6 ± 11.8	99.8 ± 15.6	0.34
Creatinine (mg/dL)[0.7–1.3]	10.2 ± 4.9	8.5 ± 4.2	0.14
eGFR (ml/min/1.73m^2^)	5.6 ± 2.5	6.8 ± 3.0	0.17
Vascular calcification more than one site (SVCS ≥ 1)	23(74.2%)	10(76.9%)	0.59
SVCS ≥ 3	6 (19.4%)	2 (15.4%)	0.56

*Abbreviations* BMI, body mass index; BSA, body surface area; PWV, pulse wave velocity; SBP, systolic blood pressure; DBP, diastolic blood pressure; Hb, hemoglobin; ALP, alkaline phosphatase; LDL, low density lipoprotein; CRP, C-reactive protein; eGFR, estimated glomerular filtration rate using CKD-EPI equation

### Sclerostin has a positive correlation with pulmonary hypertension in pre-dialysis ESKD patients

To assess the association between sclerostin in cardiac structural abnormalities in PD patients, we compared various echocardiographic parameters according to sclerostin level. Patients in the high sclerostin group had significantly increased LV diameters at end systole and end diastole and showed a reduced ejection fraction. Higher serum sclerostin levels were also associated with eccentric myocardial remodeling, a thick myocardium, and pulmonary artery hypertension, with significantly elevated pulmonary artery pressure (Table 2, Supplement Table 2).

**Table 2:** Echocardiographic parameters according to the sclerostin

Variables	Low Sclerostin (< 218.18pmol/L) *n* = 31	High Sclerostin (≥ 218.18pmol/L) *n* = 13	*P*
LVEDD (mm)	49.2 ± 7.2	54.6 ± 7.9	0.01
LVESD (mm)	31.9 ± 8.4	37.9 ± 9.3	0.01
SWT (mm)	10.4 ± 1.4	11.9 ± 2.2	0.04
PWT (mm)	10.5 ± 1.2	11.8 ± 2.3	0.04
LAVI (ml/m^2^)	32.0 ± 8.3	42.9 ± 14.3	< 0.01
LVMI (g/m^2^)	116.1 ± 31.9	152.1 ± 33.3	< 0.01
RWT (mm)	0.43 ± 0.1	0.45 ± 0.1	0.34
LVEF (%)	54.2 ± 10.2	47.3 ± 14.1	0.03
E (cm/s)	90.7 ± 97.8	83.2 ± 26.2	0.35
A (cm/s)	77.9 ± 24.0	77.9 ± 11.9	0.49
DT (ms)	205.8 ± 56.4	168.3 ± 29.0	0.02
e’ (cm/s)	6.9 ± 2.0	4.9 ± 1.7	< 0.01
a’ (cm/s)	9.0 ± 2.4	8.1 ± 2.0	0.11
s’ (cm/s)	6.9 ± 2.2	5.9 ± 1.8	0.08
RV s’ (cm/s)	11.2 ± 2.5	10.9 ± 2.3	0.35
E/e’ ratio	13.7 ± 12.4	17.7 ± 4.3	0.13
Estimated pulmonary artery systolic pressure (mmHg)	30.4 ± 6.7	44.2 ± 11.2	< 0.01
LV GLS	-15.0 ± 1.9	-15.33 ± 6.5	0.91
Pulmonary hypertension (> 35mmHg)	7(22.6%)	12(92.3%)	< 0.01

*Abbreviations* LVEDD and LVESD, left ventricular end diastolic and systolic dimension; SWT and PWT, septal and posterior wall thickness; RWT, relative wall thickness; LVMI, left ventricular mass index; LAVI, left atrial volume index; EF, ejection fraction; e’ and a’, septal early and late mitral tissue velocity; s′, septal systolic mitral tissue velocity; E and A, early and late diastolic mitral inflow velocity; GLS, global longitudinal strain; DT, deceleration time

**Fig. 1:**
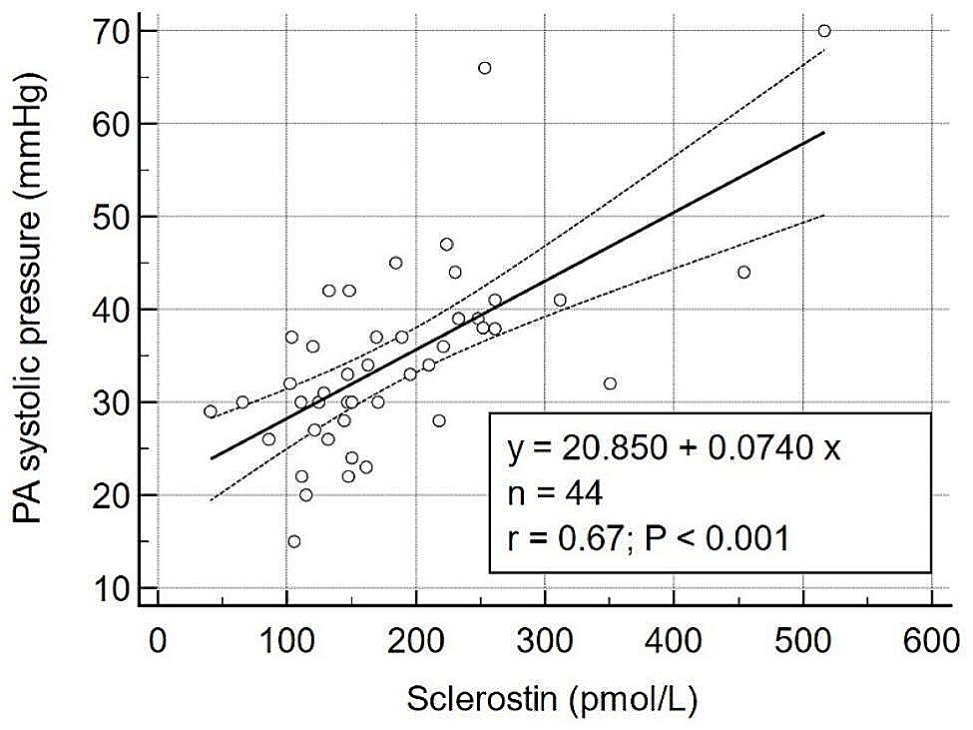
Correlation between sclerostin and pulmonary artery pressure. The sclerostin and pulmonary artery pressure had statistically significant correlation with correlation coefficient 0.67 (*p* < 0.001)

We further analyzed whether the higher sclerostin could be a risk factor for PH because of the significant association between PH and sclerostin. In the multivariable backward stepwise linear regression analysis adjusted for age, sex, body mass index, and diabetes mellitus, sclerostin was an independent risk factor of PH (Table 3). In addition, sclerostin and PA pressure showed a significant positive correlation (*r* = 0.67, *p* < 0.001) (Fig. 1).

**Table 3:** Multivariate logistic regression analysis of risk factors associated with pulmonary hypertension

Factors	Odds Ratio (95% CI)	*P*
Gender	1.56 (0.27–8.89)	0.62
Age	0.98 (0.93–1.04)	0.98
DM	3.94 (0.34–46.08)	0.28
HTN	1 (0.99–1.01)	0.98
BMI	0.88 (0.70–1.11)	0.29
SVCS ≥ 1	5.11 (0.48–54.08)	0.18
Sclerostin	41. 14 (4.53-373.89)	< 0.01

*Abbreviations* BMI, body mass index; DM, diabetes mellitus; HTN, hypertension; SVCS, simple vascular calcification score. multivariate logistic regression including age, gender-female, BMI,, SVCS ≥ 1, DM, HTN, sclerostin over 218.19 pmol/L, backward method

### Sclerostin can predict new-onset CV events and mortality

During the median follow-up period of 54.8 months, 10 patients (22.7%) developed new-onset CV events, and multivariate Cox regression analysis adjusted for sex, age, diabetes, hypertension, BMI, and the presence of PH demonstrated that sclerostin could not predict new-onset CV events (OR 2.69, 95% CI 0.89–8.13, *P* = 0.08) (Table 4).

**Table 4:** Multivariate Cox regression analysis of sclerostin and new onset CV event (Age, gender-female, DM, HTN, CAD, BMI, sclerostin over 218.18 pmol/L, PH; pulmonary hypertension– pulmonary artery pressure > 35mmHg, backward method, Abbreviations: DM; diabetes mellitus, HTN; hypertension, CAD; coronary artery disease, BMI; body mass index)

Factors	Odds Ratio (95% CI)	P
Gender	2.09(0.68–6.46)	0.20
Age	1.01 (0.98–1.06)	0.43
DM	1.27(0.22–7.34)	0.79
HTN	1.76(0.12–25.70)	0.68
CAD	1.89(0.43–8.38)	0.40
BMI	0.86(0.71–1.05)	0.15
PH (> 35mmHg)	2.35(0.37–14.92)	0.36
Sclerostin	2.69(0.89–8.13)	0.08

**Fig. 2:**
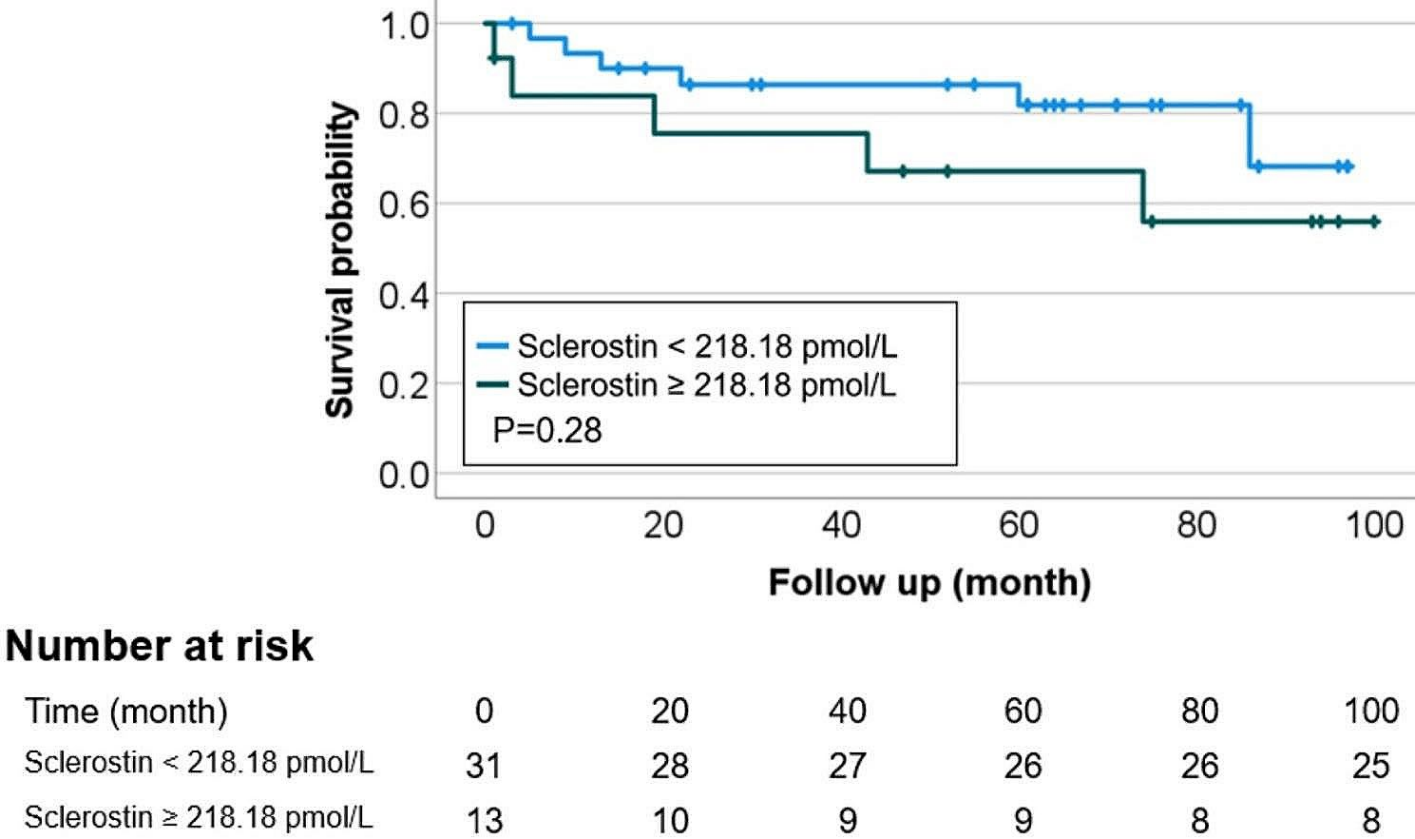
Survival graph according to sclerostin using Kaplan–Meier method. Sclerostin did not show a significant effect as a risk factor for mortality (*p* = 0.28)

Total 11 patients expired during the follow up, the mortality rate was 25%; 7 cases of sepsis, 2 cases of myocardial infarction, 1 sudden cardiac arrest, 1 case of hematochezia. and the Kaplan–Meier curve showed that the survival probability was not different between two groups. In the multivariate Cox regression analysis adjusted for age, gender-female, PH; pulmonary artery pressure > 35mmHg, systemic vascular calcification score (SVCS) over 1 and higher sclerostin over 218.18 pmol/L, the PH and SVCS were significant risk factors for all-cause mortality (Fig. 2; Table 5).

**Table 5:** Multivariate cox regression analysis for all-cause mortality (Age, gender-female, sclerostin over 218.18 pmol/L, PH; pulmonary hypertension– pulmonary artery pressure > 35mmHg, SVCS; systemic vascular calcification score over 1, backward method)

Factors	Multivariable (backward)	
	HR (95% CI)	P
Age	1.94 (0.92–1.98)	0.36
Gender	11.79 (0.49–6.57)	0.38
**SVCS**	**11.48 (2.48–53.14)**	**0.002**
**PH (> 35mmHg)**	**5.47 (1.30-23.06)**	**0.02**
Sclerostin over 218.18 pmol/L	2.70 (0.45–16.12)	0.28

The statistically significant factors were marked bold

## Discussion

In this study of 44 patients with ESKD who were predialysis ESKD PD candidates, we observed that a high sclerostin (≥ 218.18 pmol/L) level was associated with pulmonary hypertension and deleterious echocardiographic parameters. Higher sclerostin was also associated with increased mortality. This is the first study to suggest that sclerostin is an important link to pulmonary hypertension and has positive relationship with all-cause mortality beyond an altered bone–vascular axis in pre-dialysis ESKD patients.

In this study, the average sclerostin was 184.5 ± 93 pmol/L (40.9–516.7). Mödder et al. reported the serum sclerostin levels in healthy men 33.1 ± 1.0 pmol/L and healthy women 23.7 ± 0.6pmol/L [19]. They found that serum sclerostin levels was higher in elderly men compared with young women, so the highest sclerositn level was 45.1 ± 12pmol/L in elderly men. Sclerostin was elevated in the DM patient, in that study the patients non-DM healthy control group’s sclerostin was 42.11 +- 156.23 pmol/L [20]. However, this was not the decrease of the renal clearance. Renal sclerostin clearance using urinary sclerostin excretion increased with declining eGFR [21]. Sclerostin serum level elevation in CKD patients is due to overproduction, not the consequence of decreased renal elimination [22]. Compared with this, serum sclerostin significantly increased in our pre-dialysis ESKD patient group, which shows the same context as the previous studies. Although higher sclerostin levels are associated with older age, male sex, and diabetes in several other studies, we observed no differences between groups, which might because of the small number of patients.

We also observed that various cardiac parameters on echocardiography, including LV end diastolic and end systolic diameters, LV ejection fraction, and estimated pulmonary artery pressure, were significantly elevated in the higher sclerostin group. Furthermore, sclerostin levels showed a significant positive correlation with pulmonary artery pressure, suggesting a possible role for sclerostin in the development of PH in these patients. PH is an under-recognized risk factor for CV mortality in patients with CKD. According to a recent retrospective cohort study of 12,618 patients undergoing right heart catheterization, the prevalence of PH in patients with CKD was significantly higher than that in those without CKD and contributed to high mortality rates [23]. In a meta-analysis, the prevalence of PH in patients with CKD/ESKD was 23% and associated with an increased risk of CV events and all-cause mortality [24]. Our study showed that serum sclerostin could be a biomarker for the diagnosis of PH. This is important because it might be useful for screening and monitoring the treatment response. Although the specific role of sclerostin in the development of vascular calcification in patients with PH cannot be drawn from our study, recent experimental data in a CKD mouse model showing aggravated vascular calcification in Sost-/- mice in which the gene encoding sclerostin was knocked out or in mice treated with anti-sclerostin antibody showed the protective role of sclerostin, playing a counterregulatory function to decrease vascular calcification. While sclerostin’s original role prevents excess bone formation, in CKD patients, it is called calcification paradox of the sclerostin because it is pathologically increased along with an increase in osteoporosis [13, 25–27]. Still, Mare et al. concluded that the role of sclerostin as a vascular calcification predictive marker remain controversial [25].

Based on our data showing a close association between sclerostin and various echocardiographic cardiac markers, and PH, we tested the usefulness of sclerostin for predicting new-onset CV events or all-cause mortality. PH is an important disease, with high healthcare costs with 30-day re-hospitalization [28]. Bhattacharya et al. found that renal failure is a one of the risk factors to 30-day re-hospitalization [28]. It is important to predict PH, and Sclerostin may be used as one way. Of course, there is still a lot of further research needed. We observed that higher serum sclerostin levels could predict PH but it had no statistical impact on both new CV events nor all-cause mortality.

In this study we found a positive correlation between sclerostin and PH and all-cause mortality, using multivariate analysis. We provided evidence of the connection between sclerostin and PH. 54.8 months follow-up period was the longest study so far. Despite several novel findings, this study has several limitations. First, the sample size was small and selection bias might have been present. The multivariate Cox regression analysis could not find any additional role of sclerostin as a significant risk factor for mortality due to the small sample size. We also could not cover all demographic characteristics or comorbidities, including bone volume or fracture history, which may have contributed to the study outcomes. Besides most patients might have suspicious hypervolemic status according to the echocardiographic findings with increased left ventricular diastolic end diastolic and systolic dimension and E/e’ ratio. This might be representing volume overload which is risk factor for PH. One more limitations that we have to mention about the PH is the measurement method. The newer definition of PH according to ESC/ERS guidelines on PH is a mPAP > 20 mmHg. Also given the inaccuracies in estimating RAP, current recommendations are to use Tricuspid Regurgitation velocity and consider other variables to assign the probability of PH [29]. Other Right ventricular parameters such as TR jet Velocity, TAPSE and Right atrial size would also be useful. However, this study cohort has been set from 2011 to 2015, the echocardiographic data records described as 2015, 2016 recommendation which was the latest version at that time. In this study we could not provide the invasive hemodynamic results using right heart catheterization. Since PH in the setting of ESKD is a multifactorial process, thermodynamics would be helpful in separating postcapillary PH from precapillary PH. Third, the sclerostin ELISA method has not yet been standardized; therefore, we checked sclerostin levels in the experimental setting only. Lastly, we could not conclude the causal relationship between sclerostin and mortality from the pathophysiologic perspective. We only glimpse the association with sclerostin and prevalent PH, and mortality. Therefore, a large multicenter long-term follow-up clinical study and basic study are necessary to confirm our findings.

**Fig. 3:**
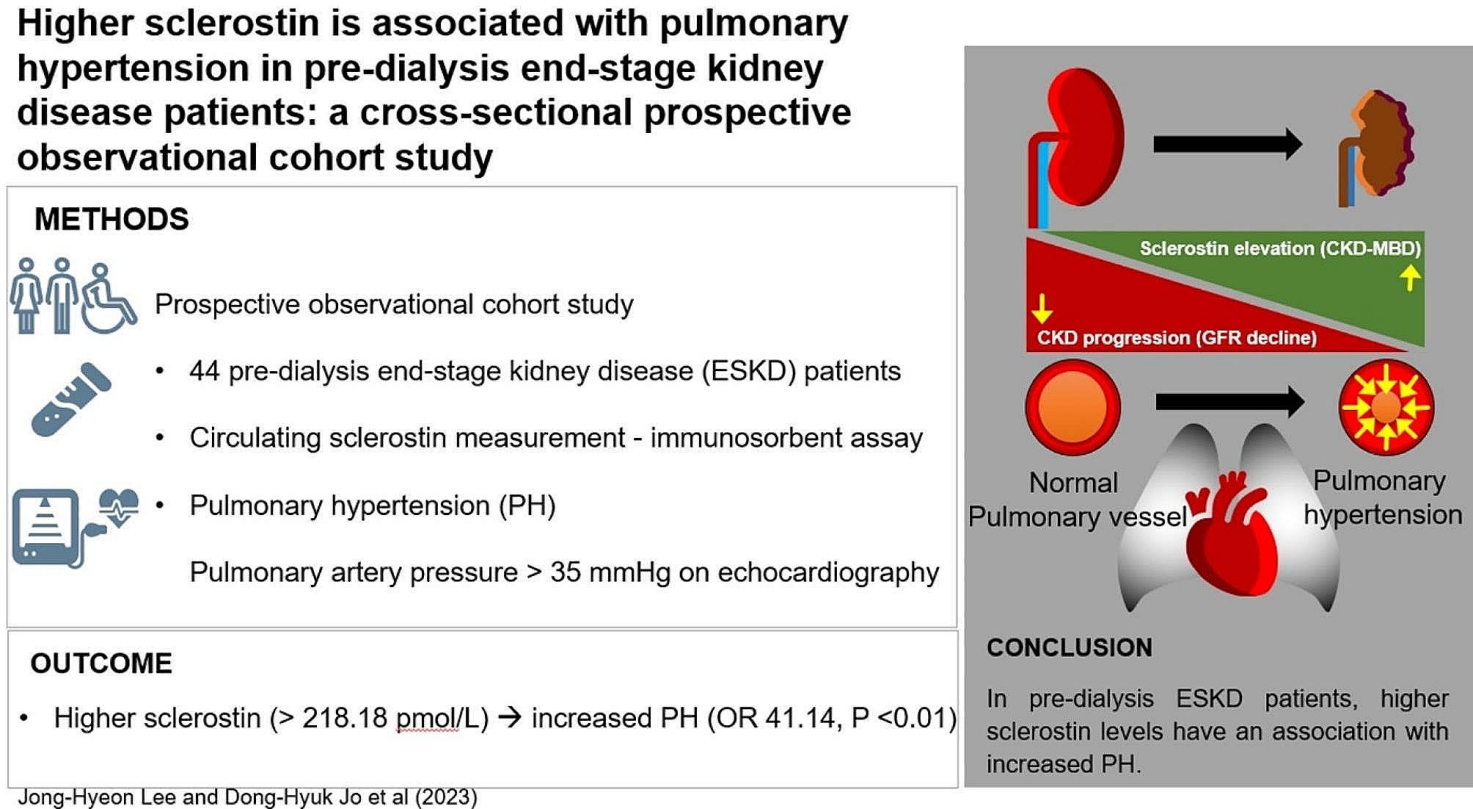
Visual abstract

Taken together, our study showed that sclerostin positive correlation with PH (Fig. 3). This supports the potentially critical role of sclerostin in CKD-MBD and the pathophysiology of PH beyond vascular calcification. Sclerostin might have a role as a molecular signal for PH, differentiate and better identify PH-left heart disease in ESKD patients.

In conclusion, this study demonstrated a higher serum sclerostin levels is associated with cardiac parameter deterioration especially PH. The higher systemic vascular calcification score and PH have an association to increase all-cause mortality in pre-dialysis ESKD patients. More studies enrolling larger numbers of patients are needed to increase our understanding of the relationship between sclerostin, CKD-MBD, and mortality in CKD patients.

### Electronic supplementary material

Below is the link to the electronic supplementary material.

Supplementary Material 1

## Data Availability

The data underlying this article will be shared upon reasonable request of the corresponding author.
