# Supplementary material for: Higher sclerostin is associated with pulmonary hypertension in pre-dialysis end-stage kidney disease patients: a cross-sectional prospective observational cohort study
Source: BMC Pulm Med. 2024 Feb 10;24:78. doi: 10.1186/s12890-024-02871-8 (PMC10858562; doi:10.1186/s12890-024-02871-8)
Supplement: Supplementary file 1 — Supplementary Material 1 [file 12890_2024_2871_MOESM1_ESM.docx]

Supplementary Figure 1. ROC curves for univariate logistic regression models of sclerostin to predict all-cause mortality


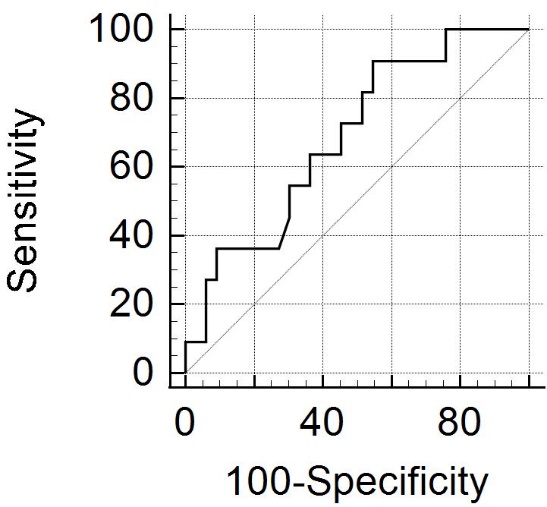


Sample size 44

Area under the ROC curve (AUC) = 0.69

Standard Error 0.09

95% confidence interval 0.53-0.82

p-value = 0.04

Associated criterion 46.05

Sensitivity 90.91

Specificity 45.45

Supplementary table 1. Correlation between sclerostin and echocardiography parameters

| SOST | LVID(D) | LVID(S) | Septum | LVPW | Aorta | LA | LAV | LAVI | LVM | LVMI | RWT | LVEF | DT | E’ | A’ | E/E’ | PApr |
| --- | --- | --- | --- | --- | --- | --- | --- | --- | --- | --- | --- | --- | --- | --- | --- | --- | --- |
| Pearson correlation | 0.294 | 0.332 | 0.336 | 0.367 | 0.240 | 0.147 | 0.370 | 0.336 | 0.434 | 0.435 | 0.069 | -0.325 | -0.325 | -0.308 | -0.081 | 0.162 | 0.665 |
| P-value | 0.053 | 0.028 | 0.026 | 0.014 | 0.116 | 0.341 | 0.013 | 0.026 | 0.003 | 0.003 | 0.656 | 0.031 | 0.038 | 0.042 | 0.623 | 0.294 | 0.000 |
| N | 44 | 44 | 44 | 44 | 44 | 44 | 44 | 44 | 44 | 44 | 44 | 44 | 41 | 44 | 39 | 44 | 44 |
